# Immutable heavy metal pollution before and after change in industrial waste treatment procedure

**DOI:** 10.1038/s41598-019-40634-2

**Published:** 2019-03-14

**Authors:** Hirokazu Ozaki, Hiroshi Ichise, Emi Kitaura, Yuki Yaginuma, Masaaki Yoda, Katsuji Kuno, Izumi Watanabe

**Affiliations:** 1 Department of Environmental Science on Biosphere, Tokyo University of Agriculture and Technology, Tokyo, Japan; 2 Western Saitama Group to Protect Soil, Water and Air, Saitama, Japan; 3 Present Address: Fukushima Branch, National Institute for Environmental Studies, 10-2, Fukasaku, Miharu-machi, Fukushima Prefecture 963-7700 Japan

## Abstract

This study compared state of pollution around an intermediate treatment plant of industrial wastes before and after the change of its treatment procedure. Bulk atmospheric deposition, surface soil, suspended particulate matter and groundwater were collected after the plant changed main operation to waste crushing and volume reduction. Their heavy metals content were comparatively investigated with the previous results obtained when it was burning wastes. The bulk heavy metals deposition showed a clear distance-related attenuation both in burning and crushing periods, indicating that the plant was the main emissions source in either case. High concentrations of heavy metals in suspended particles, soil, and groundwater during the crushing period indicated their diffusion to water environment over time. The bulk atmospheric heavy metals deposition decreased significantly, 0.20~ 0.49 times for Cu, Zn, Cd and Pb and 0.69~0.94 times for Cr, during the crushing period than burning period. However, change of their enrichment factors was not significant. It may indicate that the pollution state did not change qualitatively in a bulk deposition basis and quantitatively in a depositing particle basis. The results showed that heavy metals deposition is dominated by suspended and precipitated particulate matters that adsorb and transport the metals.

## Introduction

### Heavy metal pollution and waste treatment

Heavy metal pollution has occurred historically and worldwidely. Mining and related human activities have long been main causes of contamination since the premordern period. Especially after the industrial revolution, production activities have exhausted heavy metals and become to play important role^1–4^. Accordingly, many authors have studied its spatial distribution, impact to topsoil and vegetation around those point sources for establishing measures and estimating potential risks^5,6^.

Since the 2000s’, severe heavy metal pollution has become clear around e-waste treatment, incineration and dumping sites in developing countries due to an inadequate and/or weather-beaten procedures including crushing, separating and burning in an open field^7–10^. Regarding the contamination and waste treatment, it is not only e-wastes, but municipal and industrial wastes have been the significant causes of air, soil and water pollution around the treatment plants and dumping sites since the middle of 20th century^11–14^. That is, heavy metal pollution related to an inappropriate waste treatment technique and procedure is a big social concern both in developing and developed nations.

At the same time, heavy metal level has generally been lowered in the air in an urban residential area in the advanced countries^15–17^. The general improvement of air quality is mainly attributed from an effective regulation of atmospheric exhaust from vehicles and factories^18^. On the other hand, it is very likely that heavy metal pollution state is site-specific and largely dominated by the amount and time trend of emission from a point source. Similar to the residential area, waste treatment procedure has been altered over time based on regulations and social concern. Nevertheless, many attempts remain focusing on spatial distribution of contamination caused by mining and industrial production activities. Relatively minor number of studies^19,20^ have revealed state and distribution of pollution around waste site. Even though an effect of the countermeasure has to be examined, we do not find previous works to scientifically compare the situation before and after the measures and regulations come into force around industrial waste treatment plants as far as we reviewed previous reports. Further monitoring survey is yet required with a long-term perspective.

### Industrial waste treatment and environmental pollution in Tokorozawa, a suburban area of Tokyo

Since the late 1980s, the western part of Tokorozawa-city, Saitama Prefecture, Japan has suffered from environmental degradation caused by inadequate industrial waste treatment of many waste treatment firms. Originally, this area has commonly been referred to as “Kunugi-yama” for its suburban forest and farmland. However, its proximity to central Tokyo, approximately 30 km north-west, gave the area a serious change during the “bubble economy” period from 1980s to 1990s.

Many intermediate treatment firms of industrial waste started operation with poor facility and inadequate procedure after deforesting the suburban woods. Particularly, field burning of wastes was continued both night and day behind the suburban forest. It released enormous amount of smoke, suspended ash and offensive smell which may contain hydrogen sulfide and hydrogen chloride. Huge waste heaps appeared under a pretext of temporary storage but practically being left neglected. Flame leaped from the open waste burning caused a fire in forest and threatened people in the nearby community. A substantial traffic increase in large-sized transporting vehicle endangered pedestrians as well as a big vibrations and noise from early morning to late evening. It should also be reported that unpaved tracks in forest were covered and extended with trash and debris^21^.

Environmental condition in Kunugi-yama area was greatly degraded and seriously polluted by those inadequate treatment and disposal of industrial wastes. As a result, the safety and health conditions of residents have been adversely impacted. For example, the rate of children’s skin irritation and asthma was higher in this region than other parts of Japan, and the mortality rate of newborn babies and industrial waste burning were found to be related based on a regional survey^22^. Finally, local residents have launched campaigns against waste treatment operations and established a community association group known as the “Western Saitama Group to Protect Soil, Water and Air” in 1993 for the purpose to scientifically elucidate pollution state and remediation.

### An intermediate treatment plant of industrial waste subjected to our pollution survey

We collaboratively conducted a previous (initial) survey in Kunugi-yama area in 2001 and 2002 and showed high bulk deposition and soil concentrations of Cu, Zn, Cd and Pb near 6 industrial waste treatment plants^23^. The current study targeted one of industrial waste treatment plants (hereinafter, the treatment plant) which stopped waste burning on August 31, 2001 due to the efforts of the association group and a growing concern of the local community and society.

However, the plant was able to continue waste treatment operations as a transshipment and storage firm just in several meters from a private house but did not have sufficient measures to prevent pollution. The pollution prevention measures were not sufficient, without roof to confine contaminants in particular, both before and after the change of operation. Even after the open burning was abolished machine crushing was conducted illegally, which caused heavy noise, vibrations, malodor, and soot and dust diffusion so the environmental and residential conditions were kept worsened.

### Purpose of this study

Concern regarding environmental pollution remained after the treatment procedure was altered to a heavy crushing operation. Therefore, another on-site survey was needed to provide a successive understanding and compare the contamination state between the burning (initial) and crushing (latter) periods. Accordingly, this study was conducted to compare the heavy metals pollution state around the intermediate treatment plant during the burning and crushing periods to determine if pollutant characteristics were changed after the change in treatment procedures and to identify further tasks for environment remediation of the area.

There are many intermediate treatment firms of industrial waste keeping operation in Kunugi-yama area including the targeted treatment plant as of December 2018. Moreover, waste management is a common issue worldwide. The result of this study provides useful information to deduce pollution state, potential health risk and establishment of effective measures for suitable waste management in various countries.

## Results

### Bulk element deposition with distance from treatment plant

**Figure 1:**
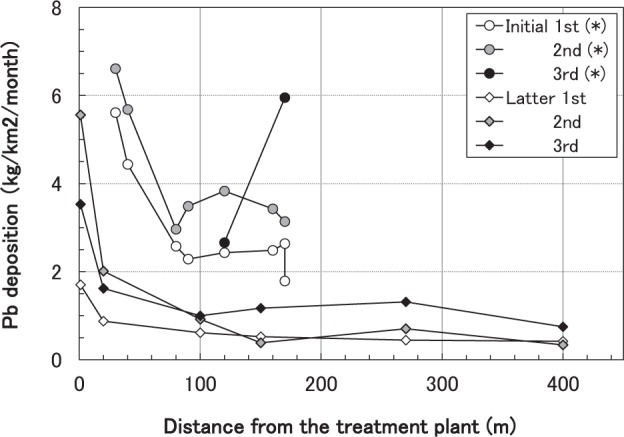
Bulk Pb deposition with distance from the treatment plant during the initial and latter sampling surveys. *Data for the initial survey are cited from Ozaki *et al*.^23^.

The initial survey in 2001 and 2002 has exhibited decreasing deposition of Al, Cr, Mn, Fe, Ni, Cu, Zn, Cd and Pb with distance from the treatment plant in the <120 m zone as reported by Ozaki *et al*.^23^. Similarly, the deposition amount of Cr, Ni, Cu, Zn, Cd and Pb decreased almost monotonically with distance from the treatment plant during the latter survey throughout its three sampling events (Table 1, Fig. 1 and Table S1). At points >100 m from the plant, the levels of the heavy metals were only 10–40% of those at the nearest point. In addition, the levels of Al, Fe, Co and Mn decreased in the <150 m zone, but then increased and peaked at south - southwest 270 m point (SSW270) (Table 1).

**Table 1:** Amount of bulk deposition of 10 elements collected from the southern side of the treatment plant in 2012–2013 (the latter survey).

Period	From the treatment plant		Bulk element deposition (kg/km^2^/month)									
	Direct-ion	Distance (m)	Al	Cr	Mn	Fe	Co	Ni	Cu	Zn	Cd	Pb
Latter 1st (*)	S	1	312	0.813	8.32	292	0.168	0.554	3.85	14.2	0.037	1.70
	S	20	205	0.336	3.98	172	0.090	0.241	1.43	5.49	0.012	0.873
	SE	100	80.8	0.176	2.76	74.7	0.038	0.123	0.858	4.45	0.011	0.612
	SSW	150	89.1	0.138	2.33	76.9	0.034	0.107	1.09	6.28	0.013	0.523
	SW	270	104	0.134	2.39	88.7	0.039	0.109	0.616	2.69	0.007	0.443
	SW	400	59.6	0.107	3.40	55.6	0.025	0.0931	0.642	3.59	0.009	0.417
Latter 2nd (**)	S	1	1600	3.27	32.2	1280	0.788	1.88	4.92	28.7	0.036	5.56
	S	20	640	1.08	11.7	513	0.277	0.595	1.92	10.2	0.014	2.01
	SE	100	611	0.713	9.63	448	0.197	0.315	1.36	5.44	0.007	0.917
	SSW	150	808	0.632	12.4	581	0.253	0.351	1.47	4.83	0.009	0.385
	SW	270	1100	0.973	16.7	776	0.333	0.517	2.05	4.61	0.011	0.702
	SW	400	673	0.51	9.95	474	0.204	0.282	1.14	2.25	0.007	0.335
Latter 3rd (***)	S	1	933	1.91	19.0	776	0.684	2.46	4.59	19.1	0.029	3.53
	S	20	602	0.968	10.8	468	0.297	0.921	2.11	8.84	0.019	1.62
	SE	100	1230	1.15	19.0	880	0.398	0.633	2.41	7.18	0.014	0.997
	SSW	150	2090	1.8	33.1	1490	0.677	1.06	4.32	7.97	0.025	1.17
	SW	270	3460	2.51	51.8	2430	1.09	1.57	5.66	11.5	0.029	1.31
	SW	400	1220	0.918	18.1	857	0.383	0.57	2.12	5.54	0.013	0.747

Periods of collection were as follows.

*June 23rd–October 16th, 2012.

**October 16th, 2012–March 11th, 2013.

***March 11th–July 18th, 2013.

It is well-known that Cr, Ni, Cu, Zn, Cd, and Pb levels in the environment are related to anthropogenic emissions. The distance-attenuation of Cr, Ni, Cu, Zn, and Cd deposition is shown around a point source; exactly those 6 elements in a mining area^24^ and Cr, Ni, Cu, Zn, and Cd around a municipal solid waste dumping site^20^. In contrast, Al, Fe, Co and Mn are generally not clearly related to human impacts because of their abundance in soil. This study demonstrated decreasing trend from the treatment plant as well for those elements in the <100 m zone. Aluminum, Cr, Mn, Fe, Ni, Cu, Zn, Cd and Pb were probably affected by the treatment plant, but the influence of soil on Al, Mn, Fe and Co relatively increased with increasing distance in the >100 m zone. The remarkable increase in Al, Mn, Fe and Co deposition at SSW270 may indicate a large amount of soil particle deposition at this location. SSW270 was located in a vegetable field; thus, it may have been more heavily influenced by soil particles than other locations. The simultaneous increase in human-derived metals deposition was probably because of their adsorption onto soil particles and co-precipitation at this location. In other words, it is likely that deposition of human-derived heavy metals such as Cu, Zn, Cd and Pb was dominated by soil particles, with the increased particle deposition being a result of the greater extent of pollutant deposition.

**Figure 2:**
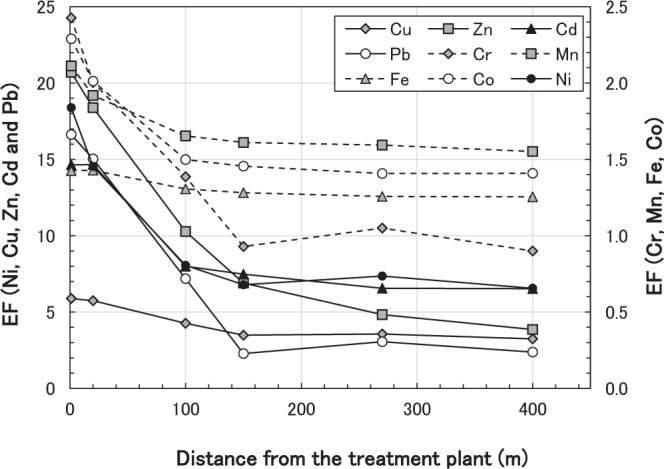
Enrichment factors of bulk heavy metal deposition at each distance to the nearest point during the second sampling of the latter survey.

These inferences were confirmed by the enrichment factor (EF_x_ = (X/Al)_amount of deposition_/(X/Al)_content in the upper crust_). The Al-normalized EF value indicates the amount of an element adsorbed on a soil particle because Al is one of main elements in soil particles (Table 2 and Table S2). Specifically, the enrichment factors of the nine heavy metals (Cr, Mn, Fe, Co, Ni, Cu, Zn, Cd and Pb) monotonically decreased with distance from the treatment plant during the latter second and third sampling events (Fig. 2) in contrast to their considerable increase of deposition (kg/km^2^/month) at SSW270. The increase in deposition was largest for Al, while it was much smaller for Cu, Zn, Cd and Pb at SSW270, which explains why the calculated EF values did not peak at this location.

**Table 2:** Al–normalized enrichment factors of the amount of bulk deposition of nine target heavy metals in 2012–2013 (the latter survey) referenced by their concentrations in the crust (†).

Period	From the treatment plant		Enrichment factor								
	Direction	Distance (m)	Cr	Mn	Fe	Co	Ni	Cu	Zn	Cd	Pb
Latter 1st (*)	S1	1	3.09	2.80	1.67	2.50	2.78	23.6	52.5	78.0	26.1
	S20	20	1.95	2.04	1.50	2.04	1.84	13.4	30.9	39.0	20.4
	SE100	100	2.59	3.59	1.65	2.16	2.38	20.3	63.6	91.7	36.2
	SSW150	150	1.84	2.74	1.54	1.79	1.88	23.4	81.3	92.8	28.1
	SW270	270	1.53	2.41	1.52	1.75	1.64	11.3	29.8	46.7	20.4
	SW400	400	2.13	5.99	1.66	1.95	2.44	20.6	69.5	102	33.5
Latter 2nd (**)	S1	1	2.43	2.11	1.43	2.29	1.84	5.89	20.7	14.6	16.6
	S20	20	2.00	1.92	1.43	2.01	1.45	5.74	18.4	14.7	15.0
	SE100	100	1.39	1.65	1.31	1.50	0.81	4.26	10.3	8.00	7.18
	SSW150	150	0.93	1.61	1.28	1.46	0.68	3.48	6.90	7.48	2.28
	SW270	270	1.05	1.59	1.26	1.41	0.74	3.57	4.84	6.56	3.05
	SW400	400	0.90	1.55	1.26	1.41	0.66	3.24	3.86	6.53	2.38
Latter 3rd (***)	S1	1	2.43	2.14	1.48	3.41	4.12	9.42	23.6	20.3	18.1
	S20	20	1.91	1.88	1.39	2.29	2.39	6.71	16.9	20.2	12.9
	SE100	100	1.11	1.62	1.27	1.50	0.81	3.75	6.74	7.52	3.88
	SSW150	150	1.02	1.66	1.27	1.51	0.79	3.96	4.40	7.75	2.68
	SW270	270	0.86	1.57	1.25	1.46	0.71	3.13	3.84	5.46	1.81
	SW400	400	0.89	1.56	1.25	1.46	0.73	3.33	5.24	7.21	2.93

^†^Element concentrations in the crust are from Lide^36^ and Taylor and McLennan^37^.

*June 23rd–October 16th, 2012.

**October 16th, 2012–March 11th, 2013.

***March 11th–July 18th, 2013.

The monotonic decline in EF indicates a decrease in the amount of heavy metals with distance from the treatment plant on a depositing particles basis. Based on the results presented above, the treatment plant was a significant emission source of heavy metals pollution in both the initial and latter periods.

### Concentration and spatial distribution of heavy metals in the surface soil

Average concentrations of Cr, Mn, Ni, Cu, Zn, Cd and Pb in soil samples collected in the latter (<2.0 cm) survey are shown in Table 3. Those from the initial survey (<15 cm)^23^ and reference non-contaminated levels in Japanese soil^25^ are provided in Table S3 (the initial survey). The concentrations of the seven heavy metals in the soil samples were 1.5–2.1 times higher for Cr, Mn, Ni and Cd and 2.3–3.4 times for Cu, Zn and Pb compared with the reference level. The Al, Fe and Co concentrations exceeded the reference levels as well. Similarly, the highest enrichment factors for Zn, Cd and Pb in the soils exceeded 9, while they were more than 3 for Ni and Cu, and 1.5–2 for Mn, Fe, Co and Mo. These EF values indicate that the surface soil around the treatment plant has been contaminated by the former five heavy metals.

**Table 3:** Element concentrations in soil samples collected in 2012–2013 (the latter survey).

Period	From the treatment plant		Concentration in soil (mg/kg)									
	Direction	Distance (m)	Al	Cr	Mn	Fe	Co	Ni	Cu	Zn	Cd	Pb
Latter (*)	S	0.1	87300	101	1350	68900	31.9	54.6	161	1150	0.892	105
	S	1	86200	95.0	1370	69500	31.9	51	151	415	0.824	93.8
	S	5	97800	81.7	1480	77800	34.9	43.2	142	212	0.521	49.3
	S	10	92400	77.2	1420	75400	34.7	46.6	138	210	0.588	45.6
	S	15	96700	79	1440	77500	34.5	43.1	134	195	0.476	42.3
	S	20	94000	70.7	1440	75200	34.2	40.9	129	183	0.435	43.3
	S	40	93700	85.9	1390	76400	32.8	45.5	134	181	0.641	42.0
	S	60	93500	88.1	1460	79700	34.2	46.2	130	145	0.617	35.6
	S	80	93400	89.5	1470	79900	33.8	47.5	123	153	0.649	38.7
	S	100	93600	93.5	1450	75400	32.2	44	130	288	0.66	42.5
	S	200	98000	118	1580	77200	32.2	57.9	146	229	0.951	57.6
Non-contaminated level (**)			73000	56	930	27	15	24	30	88	0.27	20

*Soil samples were collected from surface to <2 cm in the latter survey.

**Non–contaminated levels are shown in 2 effective digits by Takeda *et al*.^25^.

Higher heavy metals concentrations were observed in soils collected at nearer points in both the initial and latter surveys (Table 3 and Table S3). The distance-attenuation was 80 m for Cu and Zn, 20 m for Cr, Ni and Cd, and 60 m for Pb. When compared with the nearest point, the Zn level was reduced to 13.3%, Pb was 33.9%, Cd was 48.8%, and Cr, Ni, Cu were 70–76% of the nearest values at the end of lowering. A lot of previous reports have shown that heavy metals concentration decreases with distance from a point source in mining area^26–28^. The current study, moreover, demonstrated that topsoil is contaminated with heavy metals even outside the treatment facility because of their diffusion from the treatment plant and deposition and retention in the surface soil outside of the facility.

### Interrelationships between element concentrations of suspended particulate form

The element concentrations of suspended particulate form (ng/m^3^) are shown in Table 4. All pairs between Cr, Mn, Ni, Cu, Zn, Cd and Pb concentrations in PM_2.5_ were positively and significantly correlated (Mann-Whitney rank correlation test, *p* < 0.01). Pairs between Al, Cr, Mn, Fe, Co each other, except that of Al and Cr, had positive and significant relationships as well (Mann-Whitney rank correlation test, *p* < 0.05). Al, Fe and Co were positively but non-significantly (*p* > 0.05) associated with Ni, Cu, Zn, Cd and Pb respectively. Similar positive relationships were observed in PM_10_, while only Al, Cr, Mn, Fe, Co, and Ni were found to be related in TSP.

**Table 4:** Element concentrations in suspended particulate matters (ng/m^3^) in the atmosphere collected every 7–8 days between May 23 and July 18, 2013.

Fraction		Al	Cr	Mn	Fe	Co	Ni	Cu	Zn	Cd	Pb
PM2.5	Avr.	172	0.54	4.60	132	0.07	0.53	1.59	14.4	0.05	2.26
	Med.	143	0.49	4.13	125	0.06	0.39	1.34	13.7	0.04	1.61
	Min.	56.1	0.33	3.23	69.2	0.03	0.21	0.95	5.87	0.02	1.24
	Max.	377	0.83	6.75	246	0.12	1.11	2.75	23.5	0.11	4.70
PM10	Avr.	367	1.21	9.77	347	0.18	1.02	5.24	34.2	0.07	3.31
	Med.	338	1.16	9.30	340	0.18	0.88	4.99	33.6	0.06	2.65
	Min.	133	0.68	5.68	176	0.08	0.44	3.16	17.4	0.03	1.92
	Max.	735	1.99	14.8	609	0.27	1.78	7.92	64.7	0.14	6.29
TSP	Avr.	698	2.06	16.5	630	0.35	1.57	9.05	46.7	0.10	4.16
	Med.	668	2.01	16.0	623	0.34	1.43	8.79	46.0	0.08	3.50
	Min.	281	1.27	9.40	336	0.15	0.79	5.55	26.5	0.05	2.47
	Max.	1390	3.29	26.5	1090	0.64	2.62	13.5	84.2	0.19	7.38

Average, median, minimum and maximum data shown in this table was calculated from weekly raw data.

Approximately half of the Ni, Zn, Cd and Pb (42.6, 49.0, 67.8 and 61.1%) and 26% of the Cu was distributed in PM_2.5_ out of the TSP. Previous studies^29–31^ have shown that there were higher concentrations in finer particles for anthropogenic heavy metals. This consistency between the present and previous studies further indicates that the treatment plant is a probable emission source.

### Heavy metals concentrations in groundwater sample

Table 5 shows the element concentrations in the groundwater sample collected at the NE 10 m point. Zinc and Cd were present at particularly high concentrations of 1760 and 0.126 µg/L, respectively, while their reference levels in groundwater are estimated to be 19.6 and 0.06 µg/L according to the average values of Bowen^32^, Langmuir^33^, Ledin *et al*.^34^ and Leung and Jiao^35^. Furthermore, the Zn limit for aquatic organism protection is set to 30 µg/L in fresh water in Japan (NOEC: non-observed effect concentration) as well as Canada (LOEC: lowest-observed effect concentration).

**Table 5:** Element concentrations in groundwater samples collected on June 23, 2012.

Concentration (µg/L)									
Al	Cr	Mn	Fe	Co	Ni	Cu	Zn	Cd	Pb
N.A.	0.273	1.40	207	0.091	0.863	1.62	1760	0.126	0.199

## Discussion

### Comparison of heavy metals pollution state between the initial and latter surveys

#### Age difference of heavy metals deposition

**Figure 3:**
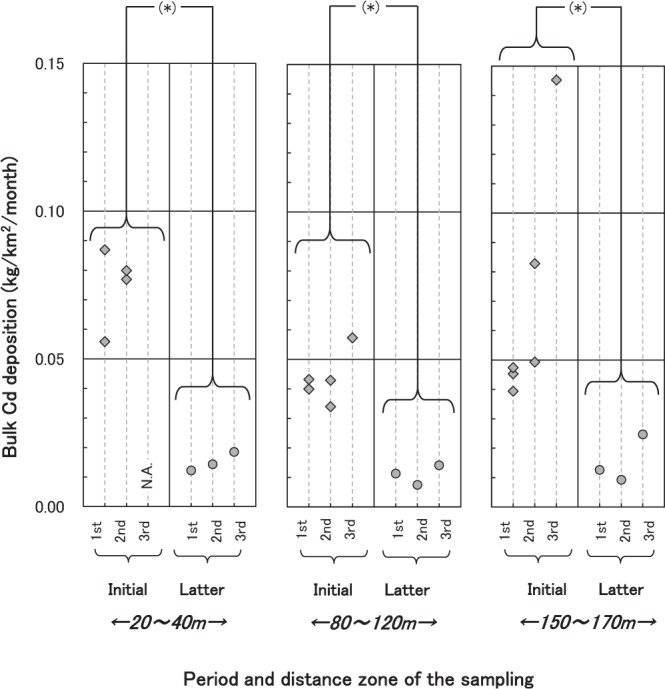
Time trend of bulk Cd deposition in the 20–40 m, 80–120 m and 150–170 m zones. Asterisks indicate significant differences between periods calculated by Mann-Whitney’s rank correlation test, *p* < 0.05.

The amounts of heavy metals deposition were compared between the initial and latter periods based on the corresponding distance zones (20–40 m, 80–120 m and 150–170 m). Bulk Cr, Cu, Zn, Cd and Pb deposition was significantly smaller in the latter survey than the initial one in the 20–40 m zone (Mann-Whitney U test, *p* < 0.05; Fig. 3). The largest and smallest Al, Mn, Fe, Co and Ni deposition showed decreasing trend from the initial survey to the latter survey (the both periods consisted of three sampling events respectively) in this zone, although they did not differ significantly between the periods.

In addition to the 20–40 m zone, Zn, Cd and Pb deposition decreased significantly in both the 80–120 m and 150–170 m zones during the latter survey when compared with the initial study. As for Cr and Cu, the smallest and largest deposition were decreased during the latter survey in the 20–40 m zone for Cr and all three zones for Cu despite being non-significant between the periods. Therefore, it can be assumed that their bulk deposition tended to decrease with time. Chromium, Cu, Zn, Cd and Pb have already been confirmed to be the main contaminants around the treatment plant in the initial survey^23^. In contrast, Al, Mn and Fe, soil-abundant elements, their smallest and largest deposition amount increased in the latter survey in all the 3 zones. Therefore, it is likely that the treatment plant strongly affected the element levels in the surrounding areas.

The initial survey^23^ was conducted in 2001 and 2002. It was when the treatment plant had been burning waste with careless procedure and then stopped burning in August 2001. Since then, the plant has been crushing solid industrial wastes for volume reduction and sorting and re-exporting waste. The latter survey was conducted approximately 11 years after the initial survey. Therefore, the change in treatment processes is a likely factor associated with decreases in heavy metals deposition, as well the almost constant deposition of soil-rich elements caused by soaring of soil particles from both the ground and soil attached to the waste.

#### Enrichment factors in the initial and latter surveys

**Figure 4:**
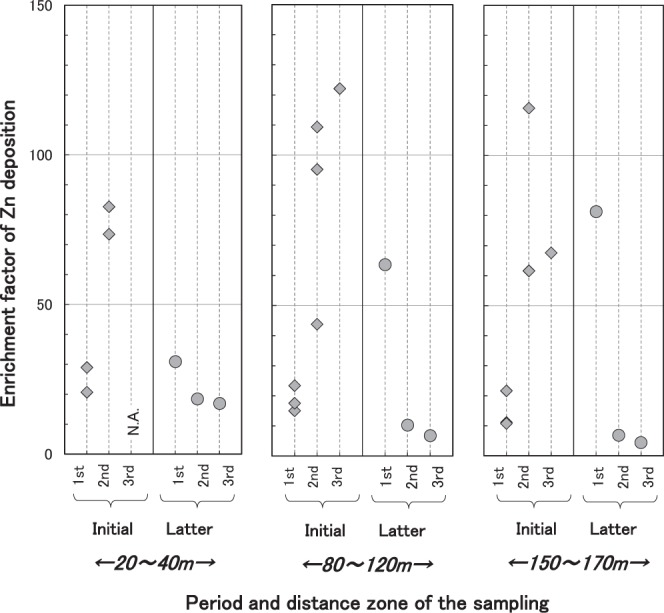
Time trend of Zn enrichment factor in the 20–40 m, 80–120 m and 150–170 m zones.

The enrichment factors for Cr, Mn, Fe, Ni, Cu, Zn, Cd, and Pb were largest in the initial second sampling and decreased in the initial third sampling and latter surveys (Fig. 4). In the 20–40 m zone, the transect EF average was <2 for Cr, Mn, Fe and Ni, 2.2 for Co, 7.7 for Cu, 26–28 for Zn and Pb, and 54 for Cd in the initial first sampling, while that in the initial second sampling was <2 for Fe and Co, 2–3 for Cr, Mn and Ni, 22 for Cu, 82 for Zn, 114 for Cd and 65 for Pb (Table 2 and Table S2). Therefore, the ratio of the average EF in this zone during the initial second to the first sampling was approximately 0.7 for Co, 1.2 for Fe and Mn, 1.6 for Ni and Cr, and >2 for Cu, Zn, Cd and Pb. Elements with a larger ratio were generally more closely related to anthropogenic factors. By the third sampling of latter survey, the enrichment factors for Fe, Cu, Zn, Cd and Pb had decreased to below those in the initial first sampling. Indeed, the EF values in the latter third sampling for Zn, Cd and Pb in the 20–40 m zone had decreased to half those obtained in the initial first sampling. The extent of the decrease in EF was larger for Zn, Cd and Pb than for Cr, Mn, Fe and Cu which showed very slight decreases.

Similar time fluctuations were observed in EF for Cr, Mn, Ni, Cu, Zn, Cd and Pb in both the 80–120 m and 150–170 m zones to that in the 20–40 m zone (Fig. 4). The initial second sampling was performed between June 23 and October 6 of 2001, which corresponded to a period when the treatment plant stopped burning waste and the furnace was dismantled. These facts imply that the widespread adverse effects in the initial second sampling may have been because of an increase in burning amount immediately prior to closure of the furnace.

Between the initial and latter surveys, the EF values for Cd and Pb in the 20–40 m zone decreased significantly (Mann-Whitney U test, *p* < 0.05), but those of others did not. Conversely, the bulk deposition amount (kg/km^2^/month) decreased significantly in the latter period for most cases (Mann-Whitney U test, *p* < 0.05; Section 2.1). The EF is a normalized value of element concentration against that of Al, which is very abundant in soil; therefore, EF can be an indicator of the amount of elements transported by adsorption onto soil particles. Accordingly, the decrease in heavy metals deposition during the latter period may be attributed to a reduction in particle precipitation. In other words, the amount of particle deposition may be directly linked to the extent of heavy metals contamination. These findings indicate an almost constant pollution state for most contaminants from the initial to latter periods on a particle basis. Overall, the results indicate that the change in operation had a very limited effect on pollution.

Emission of large sized particles may have increased as the treatment plant’s main operation changed from burning to crushing. Large particles are more quickly deposited near their source than small particles; therefore, the spatial distribution of particle deposition could have changed, with more particles being deposited in the nearer zone and less in the further zones during the latter survey. This presumption was confirmed by the distance-deposition relationship (Fig. 1), which revealed larger deposition in the <20 m than the 20–40 m zone.

### Transport of treatment plant-derived elements from atmosphere to soil and groundwater

Particulate matter (PM_2.5_, PM_10_, and TSP), bulk deposition and soil samples showed very similar orders of magnitude of their enrichment factors, with the largest being observed for Cd and Zn followed by Pb and Cu. The levels of Cd and Zn were particularly high throughout the three kinds of the environmental media. Zinc is one of the largest consumed nonferrous metals in Japan and this is maybe the reason why Zn level in the environment is clearly associated with anthropogenic activities and contamination in general. Cadmium tends to coexist with Zn from their geochemical properties. In addition, those two elements are more water-soluble compared with Cu and Pb. The commonly high Cd and Zn level from the bulk deposition to groundwater via soil is probably because of those general characteristics.

**Figure 5:**
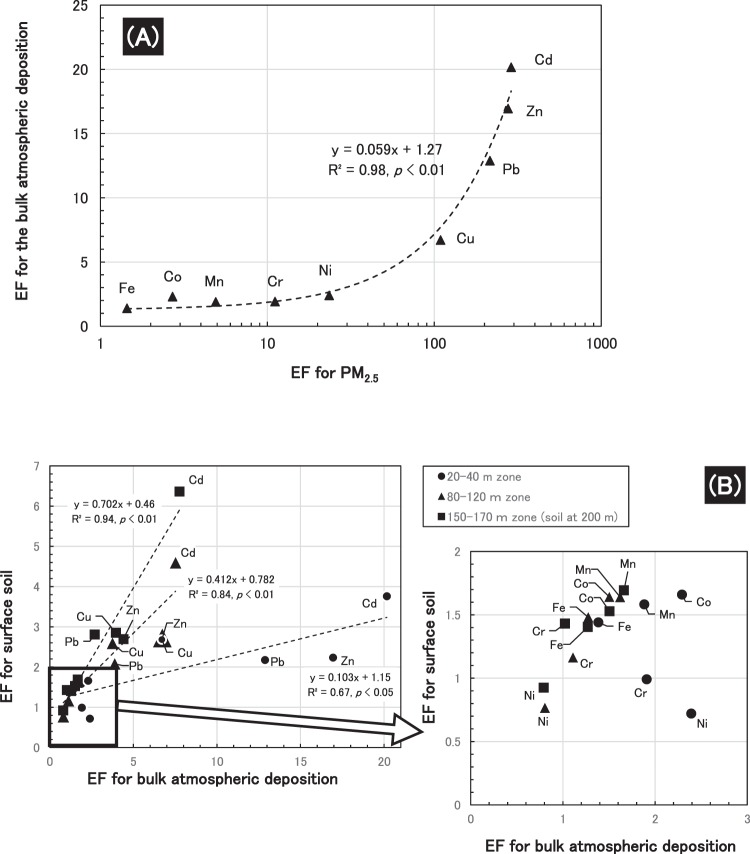
Relationship of element compositions based on EF values between. (**A**) PM_2.5_ and bulk atmospheric deposition during the latter third sampling period. (**B**) Bulk atmospheric deposition and surface soil.

The metals compositions of the three types of samples were compared by scatter diagrams of their EF values (Fig. 5) and raw measured value (concentration and deposition amount) between [1] suspended particulate matters (PM_2.5_, PM_10_, TSP) and bulk deposition (20–40 m), [2] bulk deposition and soil in each zone, and [3] soil (<10 m zone) and groundwater. The relationships ([1], [2] and [3]) in EF and concentration values revealed positive and significant correlation (Spearman’s rank correlation test, *p* < 0.01 or 0.05) (The relationship [3] was checked only based on the nine heavy metals concentrations since the concentration of Al in groundwater was not analyzed).

Overall, the common compositions indicate that the heavy metals are emitted from the same source. Furthermore, the pollution from the plant may have reached groundwater via atmospheric deposition and soil contamination based on the five positive and significant relationships (EF and concentration for [1] and [2], and concentration for [3]).

## Conclusion

This work made it clear that neighboring (20~170 m zone) of the intermediate treatment plant of industrial waste has substantially remained polluted by heavy metals. As previous workers^19,20^ did, we first focused on an amount of bulk heavy metal deposition and found its decrease when the treatment plant crushes and transships waste during the latter survey (2011–2012) relative to the burning period (the initial survey, 2001–2002). The further examination demonstrated similar levels of enrichment factors for Cr, Mn, Fe, Co, Ni, Cu and Zn between the two periods. This is probably because suspended particles are the main transporting media on which heavy metals are adsorbed. These findings give a conclusion that the change in operation of the treatment plant did not drastically contribute to qualitative reclamation. Moreover, pollution level on a depositing particle basis were quantitatively very similar degree before and after the change of operation.

The similar composition of element enrichment factors between PM_2.5_ and bulk deposition reconfirmed that particulate matter emitted by the crushing treatment was deposited on the ground after being suspended in the atmosphere. It may again indicate heavy metals deposition was associated with the particle deposition and dominated by it. Additionally, the constantly high heavy metals level in the surface soil and elevated Zn and Cd concentrations in the groundwater revealed remaining and diffusion of the heavy metals. Therefore, potential diffusion risk of the pollutants should be monitored in the long-term basis.

The Kunugi-Yama area is a part of the marginal Tokyo metropolitan region. This area name has come from its suburban forests (Satoyama). This area has been threatened by deforestation, illegal and/or inadequate waste treatment, and consequential pollution problems. Issues this study exhibited may not only and simply be a heavy metal pollution problem in a certain region in Japan. It could rather be issues of conflict between expanding economic activities and environmental conservation in a suburban area. Similar environmental contamination situations likely exist worldwide, particularly in countries that may be suffering from increasing industrial wastes. Accordingly, appropriate measures to address such types of pollution are required.

## Methods

### Bulk Atmospheric Deposition

#### Periods of sample collection

The current (the latter survey) and previous (the initial survey) studies both consisted of three sampling periods (Table 6). The latter survey collected samples between June 23 and October 16, 2012 (latter 1st sampling), October 16, 2012 and March 11, 2013 (latter 2nd sampling), and March 11 and July 18, 2013 (latter 3rd sampling). The previous three comparable samples were collected from February 4 to May 13, 2001 (initial 1st sampling), June 23 to October 6, 2001 (initial 2nd sampling), and October 6, 2001 to January 27, 2002 (initial 3rd sampling)^23^.

**Table 6:** Location, period and zone from which bulk atmospheric deposition samples were collected.

Direc- tion	Dist- ance (m)	Zoning for inter-period comparison	Initial Survey ^(*)^			Latter Survey		
			1st	2nd	3rd	1st	2nd	3rd
			Feb. 4th–May 13th, 2001	Jun. 23rd–Oct. 6th, 2001	Oct. 6th 2001–Jan. 27th 2002	Jun. 23rd–Oct. 16th, 2012	Oct. 6th 2012–Mar. 11th 2013	Mar. 11th–Jul. 18th, 2013
S	1		—	—	—	⃝	⃝	⃝
S	20	20–40 m	—	—	—	⃝	⃝	⃝
S	30		⃝	⃝		—	—	—
SSW	40		⃝	⃝	×	—	—	—
SSW	80	80–120 m	⃝	⃝	×	—	—	—
S	90		⃝	⃝	×	—	—	—
SE	100		—	—	—	⃝	⃝	⃝
SSE	120		⃝	⃝	⃝	—	—	—
SSW	150	150–170 m	—	—	—	⃝	⃝	⃝
SSW	160		⃝	⃝	×	—	—	—
S	170		⃝	⃝	⃝	—	—	—
SSW	170		⃝	×	×	—	—	—
SW	270		—	—	—	⃝	⃝	⃝
SW	400		—	—	—	⃝	⃝	⃝

*The initial survey is a part of Ozaki *et al*.^23^.

#### Procedure of collection

**Figure 6:**
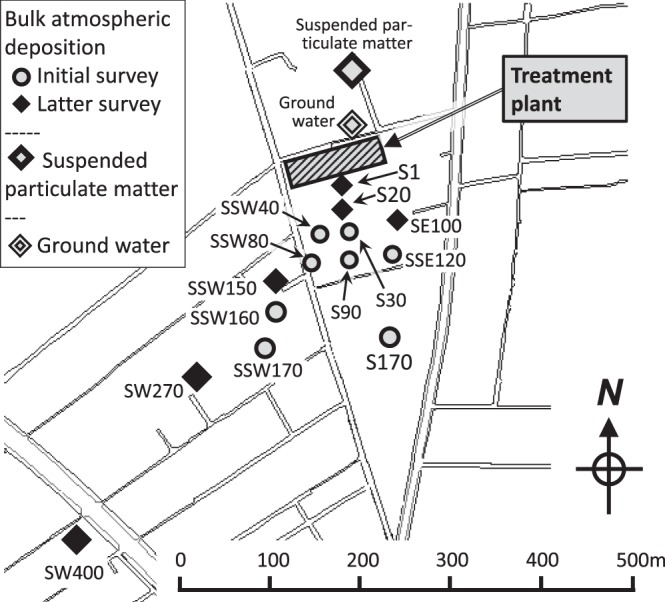
Locations of bulk atmospheric deposition, suspended particulate matter and groundwater collected during the initial and latter surveys.

Polyethylene (PE) bottles with a volume of 10 L and 10 cm opening were washed with 6% HNO_3_ and rinsed with deionized water for use as passive collectors. The washed PE bottles were installed at eight and six locations in the initial and latter surveys, respectively. All sampling locations were southeast to southwest of the treatment plant, from immediately outside the facility to 400 m-distance (Fig. 6). At the installation points, one bottle was positioned at a height of 4 m to prevent contamination of particles soaring from the ground. Both dry and wet deposition were collected together as bulk samples during the periods shown in the previous section.

#### Evaporation, concentration and element extraction

Liquid and solid mixed materials collected in the PE bottle were subjected to the following pretreatment within 7 days and element determination within 30 days. The materials were transferred to a 1 L glass beaker. The inner walls of the PE bottles were washed by 20 ml of 61% HNO_3_ (EL-grade, Kanto Kagaku Co., Ltd., Tokyo) for two times and the HNO_3_ were added to the beaker. The PE bottles were subjected to 100 rpm side-over-side shaking for 12 hours with another 20 ml of HNO_3_ and then the HNO_3_ was delivered to the beaker. Finally, ultra-pure water was used for 3 times to rinse the inner wall of the PE bottle and added to the glass beaker.

The liquid in the beaker was evaporated on a hot plate (120 °C) to approximately 20 ml. Then, the concentrate of the mixed material was subjected to complete digestion using 61% HNO_3_ and 35% HCl according to the United States Environmental Protection Agency 3015 standard method (USEPA, 2007). The digested sample was then filtered by Advantec® quantitative filter paper No. 5C (Toyo Roshi Co., Ltd., Tokyo) and weighed.

### Suspended particulate matter

#### Instruments, location and period of collection

Suspended particulate matters were collected using a low-volume air sampler (Oct Science, MF-200, Hyogo) installed about 30 m northeast of the treatment plant (Fig. 6). The air flow rate was set to 20 L/min and a Nilu filter holder (Tokyo Dylec Corp., Tokyo) was employed for cascade impactor to separate the particles into three fractions, TSP-PM_10_, PM_10-2.5_ and PM_2.5_. Zefluor™ PTFE membrane filters (Pall Corporation, Tokyo) were used to capture particles for the former two fractions and an Advantec® PF020 filter (Toyo Roshi Co., Ltd., Tokyo) was used for PM_2.5_.

The filters were continuously exchanged every 7 or 8 days from July 4, 2013 to December 5, 2013, after which they were packed in polyethylene bags and stored in a refrigerator (<10 °C) until analysis.

#### Element extraction from filters

The filters were cut to millimeter sized pieces and placed into PFA digestion vessels, after which 2.5 ml HNO_3_ (61%, EL-grade, Kanto Kagaku Co., Ltd., Tokyo), 1.5 ml HF (Guaranteed Reagent, Wako Chemical Co., Ltd., Osaka) and 0.5 ml H_2_O_2_ (Super Special Grade, Wako Chemical Co., Ltd., Osaka) were added. Then, the vessels were firmly sealed and irradiated at 200 W for 10 minutes. After cooling to room temperature, the vessels were uncapped and put on a hot plate (200 °C) to volatilize the remaining acid. Finally, 2.5 ml of HNO_3_ was added to bring the inorganic components into solution, after which the samples were passed through No. 5C filter paper. The same procedure was applied to an unused filter as a blank.

### Surface soil

Surface soils were collected from around the treatment plant as the main reservoir of pollutants deposited from the atmosphere in December 2000 and February 2001 during the initial survey^23^ and August and October 2013 during the latter survey. The collection depths and locations were <15 cm at 10, 100, 120, 200 and 300 m during the initial survey and <2 cm at 0.1 (immediately outside of the treatment plant’s premises), 1, 5, 10, 15, 20, 40, 60, 80, 100 and 200 m south of the treatment plant during the latter survey. A plastic scoop was used for sample collection to prevent metals contamination. The surface layer soil was collected from 4–5 points within several square meters in one location and mixed into one plastic bag as a composite sample to ensure the location’s representativeness.

Soil samples were ground with a mortar and pestle after being dried at 50 °C for 24 hours or more. The <2.0 mm fraction was then digested with HF-HClO_4_-HNO_3_ on a 220 °C hot plate, after which the digest was passed through No. 5C filter paper to remove the remaining particles.

### Groundwater sample

Groundwater was collected from a well located 10 m northeast of the treatment plant on June 23, 2012. The depth of the water was 31 m and that of the pump was 44 m. The water sample was immediately passed through No. 5C filter paper and then acidified to pH <2 with HNO_3_ to preserve the dissolved fraction before analysis for heavy metals.

### Determination and calculation of element concentrations

Concentrations of ^27^Al, ^52^Cr, ^55^Mn, ^56^Fe, ^59^Co, ^60^Ni, ^63^Cu, ^66^Zn, ^111^Cd and ^208^Pb in all sample solutions prepared by the above pretreatments were determined by ICP-MS (Agilent, 7500a) with 500 mg/L ^103^Rh as an internal standard using an online supply system.

The concentrations of elements in the concentrated and digested bulk atmospheric deposition samples were calculated to determine the amount deposited per area per period (kg/km^2^/month). Concentration data for suspended particulate matter samples were converted to suspended amount per air volume (ng/m^3^). The amount of bulk deposition and the concentrations in suspended particle matter and surface soil were recalculated to determine enrichment factor (EF_x_ = (X/Al)_content in the sample_/(X/Al)_content in the upper crust_, where X indicates each of the nine target elements) using the element abundance in the upper crust as proposed by Lide^36^ and Taylor and McLennan^37^.

## Supplementary information

Supplementary Dataset 1

## Data Availability

All data for in this study are included in the main and supplementary tables (Tables 2~6, Supplementary Tables 1~3).
