# Supplementary material for: Immutable heavy metal pollution before and after change in industrial waste treatment procedure
Source: Sci Rep. 2019 Mar 14;9:4499. doi: 10.1038/s41598-019-40634-2 (PMC6418243; doi:10.1038/s41598-019-40634-2)
Supplement: Supplementary file 1 — Supplementary Dataset 1 [file 41598_2019_40634_MOESM1_ESM.docx]

**Immutable heavy metal pollution before and after change in industrial waste treatment procedure**

**Authors**

OZAKI, Hirokazu^1, *)^, ICHISE, Hiroshi ^1)^, KITAURA, Emi ^2)^, YAGINUMA, Yuki ^1)^, YODA, Masaaki ^1)^, KUNO, Katsuji ^1)^ and WATANABE, Izumi ^1)^

**Authors’ affiliations**

^1)^ Department of Environmental Science on Biosphere, Tokyo University of Agriculture and Technology

^2)^ Western Saitama Group to Protect Soil, Water and Air

*) Corresponding author and current affiliation: National Institute for Environmental Studies; 10-2, Fukasaku, Miharu-Machi, Fukushima Prefecture, ZIP 963-7700, Japan, TEL +81-247-61-6114 (Ext.6567), E-mail [ozaki.hirokazu@nies.go.jp](mailto:ozaki.hirokazu@nies.go.jp)

**Table captions**

Table S1 Amount of bulk deposition of 10 elements collected from the southern side of the treatment plant in 2001–2002 (the initial survey) ^23^

Table S2 Al-normalized enrichment factors of the amount of bulk deposition of nine target heavy metals in 2001 - 2002 (the initial survey) ^23^ referenced by their concentrations in the crust (†)

Table S3 Element concentrations in soil samples collected in 2001–2002 (the initial survey) ^23^
